# Correction of vital statistics based on a proactive search of deaths and live births: evidence from a study of the North and Northeast regions of Brazil

**DOI:** 10.1186/1478-7954-12-16

**Published:** 2014-06-05

**Authors:** Célia Landmann Szwarcwald, Paulo Germano de Frias, Paulo Roberto Borges deSouza Júnior, Wanessa da Silva de Almeida, Otaliba Libânio de Morais Neto

**Affiliations:** 1 Institute of Communication and Information Science and Technology in Health, Oswaldo Cruz Foundation, Ministry of Health, Av. Brasil, 4365 – ICICT room 225 - Manguinhos, Rio de Janeiro 21040-360, Brazil; 2 Institute of Medicine Professor Fernando Figueira, Rua dos Coelhos, 300 – Boa Vista, Recife, Pernambuco 50070-550, Brazil; 3 Federal University of Goiás, Rua Delenda Rezende de Melo, s/n - Setor Universitário, Goiânia 74605-050, Brazil

**Keywords:** Vital statistics, Underreporting, Correction method, Proactive search, Infant mortality rate, Brazil

## Abstract

**Background:**

In the last 20 years, Brazil has undergone dramatic changes in terms of socioeconomic development and health care. In the first decade of the 2000s, the Ministry of Health (MoH) developed a series of programs focused on reducing infant mortality, including the Family Health Program as a national policy for primary care. In this paper, we propose a method to correct underreporting of deaths and live births. After vital statistics are corrected, infant mortality trends are analyzed for the period 2000–2010 by macro-geographical region.

**Methods:**

A proactive search of live births and deaths was carried out in the Amazon and Northeast regions in 2010 to find vital events that occurred in 2008 and were not reported to the Ministry of Health. The probabilistic sample of 133 municipalities was stratified by adequacy of vital information reporting. For each municipality, the adequacy analysis was based on the reported age-standardized mortality rate per 1,000 population and the ratio between reported and estimated live births. Correction factors were estimated by strata based on additional vital events found in the proactive search. The procedure was generalized to correct municipal vital statistics for the period 2000–2010.

**Results:**

In the proactive search, 35% of non-reported deaths were found within the health system (hospitals and other health establishments), but 28% were found in non-official sources, like illegal cemeteries. In areas of extreme poverty and unreliable vital information, the estimated completeness of infant death reporting was only 33%. After correction of vital information, the estimated infant mortality rate decreased from 26.1 in 2000 to 16.0 in 2010, with an annual rate of decrease of 4.7%, greater than the required rate to achieve the Millennium Development Goal. Among Brazilian regions, the Northeast showed the largest decrease, from 38.4 to 20.1 per 1,000 live births.

**Conclusions:**

The proactive search for vital events was shown to be a good strategy both in terms of understanding local irregularities and for correcting vital statistics. The methodology could be applied in other countries to routinely assess the pattern and extent of birth and death under-registration in order to improve the utility of these data to inform health policies.

## Background

In the last 30 years, Brazil has undergone several changes in terms of socioeconomic development, urbanization, and health care. The growth of urbanization, improvement in women’s education, greater female participation in the labor market, and the increased availability of contraceptive methods resulted in a sharp decrease in fertility, with direct and indirect effects on mortality during the first year of life [1,2].

In terms of health care, the country has adopted a unified health system, with profound changes in health care policies and a marked expansion of primary health care [3]. During these years, the Ministry of Health (MoH) developed a series of programs focused on reducing infant mortality, including the Family Health Program as a national policy for primary care, giving priority to municipalities with the worst socioeconomic levels, located in the North and Northeast [4]. Estimation of infant mortality and monitoring of temporal trends became essential in very poor areas with unreliable vital information.

The Brazilian Ministry of Health has two vital information systems: the Mortality Information System (SIM), with approximately 1.2 million annually reported deaths, and the Live Birth Information System (SINASC), with 3 million annually reported live births. Unidentified data from both systems are openly available on the Internet, aggregated by municipality.

The Mortality Information System (SIM) provides information on socio-demographic characteristics of the deceased (or the mother’s characteristics in case of an infant death), circumstances of death, and the cause of death, classified according to the International Classification of Diseases (ICD). The system was created in 1976 by implementing the standard model of the death certificate throughout the national territory. In most cases, this document is signed by a doctor and issued in triplicate. The first copy is sent to the Municipal Secretary of Health for typing and reporting to the Ministry of Health. The second copy is delivered to the family, for registration in a Civil Registry Office, while the third one is retained in the hospital. Only in places where there are no doctors, officers of Civil Registry may issue a death certificate in the presence of two witnesses. In this circumstance, the Civil Registry should send a copy of the death certificate to the Municipal Secretary of Health.

In theory, no burial should take place without a death certificate and death registration. However, in practice, irregular burials are known to occur, especially in the North and Northeast regions [5]. In a previous study, the main flaws found in the process of reporting vital events to the MoH were absence of strategies for death certification in cases of household deaths in rural regions; issue of death certificates by non-doctors; problems of transfer of local data to the national database; and lack of perception of the importance of death registration by the local community [6].

The Live Birth Information System (SINASC) was created in 1990, based on the live birth certificate, a document that must be issued at the health care facility where the delivery occurs. As 98% of deliveries in Brazil occur in hospitals, the coverage of SINASC is high. The live birth information system provides information on the conditions of birth, including birth weight and gestational age, as well as socio-demographic characteristics of the mother.

Due to death underreporting in some areas of the country, until the 1990s, indirect demographic methods based on household surveys were used to estimate the probabilities of death by age group, specifically in the first year of life. However, given the restrictions on the use of mortality estimates based on sample surveys [7-9], efforts have been made to improve the two MoH vital information systems.

Methods have been proposed to evaluate the information about deaths and births using indicators to evaluate completeness and regularity of information at the municipality level [10-12]. Other methods were based on linkage procedures of the health information systems [13-15].

Various government initiatives have been adopted to improve completeness and quality of vital information, such as strategies to reduce the number of ill-defined deaths; use of other health information systems, such as the Hospitalizations Information System (SIH) and the Primary Care Information System (SIAB), to find vital events not reported in the vital information systems; establishment of goals to increase the completeness of death reporting; and implementation of committees to investigate infant and maternal deaths across the country [4].

Moreover, research projects have been developed specifically to detect vital events unknown to the health system. The active search for deaths and births was encouraged, and some studies were carried out at the beginning of the 2000s to find events not reported to the MoH in specific municipalities with very poor vital information [6,16,17].

From September 2009 to June 2010, a proactive search of live births and deaths was carried out in the Amazon and Northeast regions to find vital events that occurred in 2008 and were not reported to the MoH. Because of the greater underreporting of deaths in those two regions, a probabilistic sample of 133 municipalities located in the 17 states that make up these regions was selected. Correction factors for birth and death statistics were estimated for this year based on the additional vital events found in the proactive search [18].

In this paper, the method was generalized to correct information on total and infant deaths and live births in the period 2000–2010. After correcting the vital statistics, we analyzed the changes in completeness of vital information reporting and in infant mortality in Brazil by macro-geographical region.

## Methods

Brazil is politically and geographically divided into five distinct regions (North, Northeast, Southeast, South, and Center-West) with varied physical, demographic, and socioeconomic characteristics. The North and the Northeast regions have the worst levels of socioeconomic development and completeness of death reporting.

The country is composed of a federal district and 26 states that are subdivided into 5,565 municipalities. The population size of each municipality varies widely: the smallest municipality has 805 inhabitants, and the largest, São Paulo, has over 11 million.

The “Proactive Search for Deaths and Live Births in the Legal Amazonia and the Northeast Regions” was carried out from September 2009 (after the 2008 live births and mortality information systems were closed) to June 2010. The research was approved by the Research Ethics Committee of the Oswaldo Cruz Foundation.

A probabilistic sample of 133 municipalities located in 17 states of the Legal Amazonia and Northeast regions was selected. The eight states that make up the Legal Amazon and the nine states of the Northeast region contain 37.7% of the total Brazilian population (9.9% and 27.8%, respectively).

The sample was stratified by the population size of the municipalities (1 to 20,000 inhabitants; 20,001 to 50,000 inhabitants; 50,001 to 200,000 inhabitants; more than 200,000 inhabitants) and by the adequacy of the vital information (deficient; unsatisfactory; satisfactory). The criteria for classifying the adequacy of the vital information have been proposed previously [7]. All of the state capitals were included in the survey.

In the sampled municipalities, we carried out a proactive search process of vital events that occurred in 2008 to identify live birth and death certificates issued but not reported to the MoH vital information systems, as well as live births and deaths whose certificates were not issued. The following sources of information were used: registry offices; Unified Registry of the Federal Government Social Programs; hospitals; primary health care units; death investigation services; institutes of forensic medicine; patient transportation services; official and unofficial cemeteries (burial sites); funeral homes; and traditional midwives. The proactive search was carried out in registry offices and hospitals located in the sampled municipality and in neighboring municipalities, where births and deaths of the sampled municipality residents are likely to occur.

Data collection was performed with a standardized instrument and encompassed all births and deaths, including fetal and non-fetal deaths, of residents in the selected municipalities that occurred between January 1 and December 31, 2008. Fetal deaths were included in the study to enable further validation of the type of death.

To carry out the fieldwork, the Health Surveillance Secretary of the MoH provided a nominal list of all births and deaths in 2008 of residents in the selected municipalities. The list was drawn from births and deaths reported to SINASC, SIM, and the Hospitalization Information System (SIH). Data gathered locally in the active search process were used to complete the original list of deaths and births.

The deaths or births found in the proactive search that were not reported to the health information systems SIM, SINASC, and SIH, not recorded in Civil Registry offices, and not found in primary health care units were confirmed through household interviews. Many of the addresses were located with the help of community health workers or at local primary health units, in general in less than two weeks after the end of the active search. In the case of an infant death or live birth confirmation, the interview was carried out with the child’s mother. In the case of deaths among people aged one year and over, the interview was conducted with a household member who could provide information about the deceased, after the informed consent. The questionnaire was composed of all variables used to fill the death certificate form or to fill the birth certificate form, according to the event to be confirmed.

The vital statistics correction factors for underreporting in the sampled municipalities were based on the additional data obtained through the proactive search. Underreporting correction factors for total and infant deaths were estimated separately [18].

### Correction of the number of reported deaths

To characterize the level of completeness of death information, the age-standardized mortality rate (ASMR) was calculated by municipality. Values above 5 per 1,000 inhabitants indicate adequate death reporting while values lower than 3 per 1,000 inhabitants indicate important underreporting.

Due to the large proportion (45%) of municipalities with fewer than 10,000 inhabitants, the ASMR was calculated by triennium, by considering the average number of informed deaths every three years so that the indicator would be more stable. Therefore, we considered the trienniums 1999–2001, 2000–2002, up to 2009–2011, corresponding to the years 2000, 2001, and 2010, respectively. For each year, the Brazilian population of the same year was used as the standard population.

For the triennium 2007–2009, corresponding to the year of the proactive search, all Brazilian municipalities were categorized by macro-geographical region and municipality population size. In each category, we estimated the median age-standardized mortality rate among the municipalities with adequate mortality information (ASMR greater than 5 per 1,000 inhabitants). Then, as an indicator of the completeness of death reporting, we calculated the following ratio for each municipality: **R** = ASMR/maximum (ASMR, median ASMR in the municipality category).

To estimate the municipal death correction factors for year 2008, we fitted a log-log regression model to the active search sampled municipalities with the logarithm of the death correction as the response variable, and the logarithm of ***R*** as the independent variable [18]. The model was applied to all Brazilian municipalities to estimate the predicted total death correction factors in each municipality.

For the estimation of the municipal infant death correction factors due to underreporting, we used an additional variable based on the observed infant mortality rate (IMR) and the median IMR calculated in the corresponding category: **R**_**IM**_ = IMR/maximum (IMR, median IMR in the municipality category).

A log-log regression model was fitted to the active search sampled municipalities by considering the logarithm of the infant death correction as the response variable, and the logarithm of the total death correction factor and the logarithm of ***R***_***IM***_ as the independent variables. The predicted municipal correction factors for total and infant death underreporting were used to estimate completeness of total and infant death reporting in all Brazilian municipalities, 2008 [18].

In the triennium 2007–2009 (corresponding to 2008), all Brazilian municipalities were categorized according to the age-standardized mortality rate: <2; ≥2 and <3; ≥3 and <4; ≥4 and <5; ≥5 and <5.5; and ≥5.5 per 1,000 inhabitants. In each ASMR category, the underreporting correction factors were estimated by the ratio of the sum of the 2008 predicted number of deaths and the sum of the 2008 reported deaths. Infant and total deaths were considered separately.

To generalize the death correction procedure in the period 2000–2010, the same procedure was used in the other trienniums. For each year of the period 2000–2010, all municipalities were categorized according to the age-standardized mortality rate, and the corresponding correction factors due to total and infant death underreporting were applied. With this method, the correction factors are held constant by ASMR category but not by municipality. That is, if there is improvement in the completeness of death reporting in a given municipality over time, a smaller correction factor will be applied as it moves to another category of ASMR.

### Correction of live births

To characterize the adequacy of the live birth (LB) information, the ratio (**R**_**LB**_) between the reported and estimated LBs was calculated by municipality. The estimated number of LBs was based on the estimated population of children less than 1 year of age. A live birth ratio (**R**_**LB**_) above 0.9 indicates adequate LB information, while values less than 0.7 indicate relevant underreporting.

Similar to the mortality data correction, the average number of informed LBs per triennium was used (1999–2001 up to 2009–2011). A log-log regression model was fitted to the active search sampled municipalities with the logarithm of the live birth correction factor as the response variable, and the logarithm of ***R***_***LB***_ as the independent variable. The predicted municipal LB correction factors were used to estimate completeness of LB registration in all Brazilian municipalities, 2008 [18].

For the triennium corresponding to the year 2008, all municipalities were grouped into categories according to the live birth ratio (<0.5; ≥0.5 and <0.6; ≥0.6 and <0.7; ≥0.7 and <0.8; ≥0.8 and <0.9; and ≥0.9). In each category, the underreporting correction factors were estimated by the ratio of the sum of the predicted number of LB and the sum of reported LB.

For the correction of the number of reported live births in the period 2000–2010, in each triennium, the municipalities were classified according to the live birth ratio category. In each category, the reported live births were corrected by the corresponding LB correction factors. Similar to the correction of mortality data, if there is improvement in the completeness of LB information in a given municipality over the period, a smaller correction factor will be applied as it moves to a higher category.

To estimate the infant mortality rate (IMR) by macro-geographical region in the period 2000–2010, the corrected numbers of infant deaths and live births in all municipalities of the region were used.

## Results

Table 1 shows the deaths and live births found in the proactive search by information source. In view of the multiplicity of sources for the same event, the information sources were given the following hierarchy: hospitals; registry offices; primary health care units; death investigation services; official and unofficial cemeteries; funeral houses; government social programs; and other sources. Over 35% of the deaths were found within the health care system (hospitals and other health facilities) and 31% in the registry offices. It is worth noting that a large proportion of deaths not found in hospitals or registry offices were found in cemeteries (16.5%) and funeral homes (11.5%).

**Table 1:** Vital events found in the proactive search by information source, Amazonia and Northeast Brazil, 2008

**Source**	**Deaths**	**Live births**
	**n (%)**	**n (%)**
Hospitals	2,196 (26.4)	9,027 (51.5)
Registry offices	2,588 (31.1)	7,397 (42.2)
Primary health care units	355 (4.3)	350 (2.0)
Institutes of forensic medicine/services of death investigation	402 (4.8)	-
Official and unofficial cemeteries	1,368 (16.5)	-
Funeral homes	960 (11.5)	-
Unified Registry of the Federal Government Social Programs	83 (1.0)	578 (3.3)
Other	360 (4.3)	175 (1.0)
Total	8,312 (100.0)	17,527 (100.0)

Among the 2,811 deaths found in unofficial sources, we located 2,363 cases (84%) in Civil Registry offices and primary health care units, making it possible to confirm the year of death (2008) and the municipality of residence. Among the remaining 448 cases, 424 (94.6%) could be located by the name or address of the deceased, from which four deaths occurred in other years (one in 2006, one in 2007 and two in 2009), and 12 could not be confirmed at home (empty house). Overall, 36 (0.4%) could not be confirmed in any of the sources and were not considered in the analysis.

Of the 408 cases confirmed in households, 262 (64.2%) died at home and 100 (24.5%) occurred in health facilities. Among those who died at home, less than 20% had a death certificate. However, among the deaths that occurred in hospitals, more than 60% of the cases had a death certificate but the death was not reported to the Ministry of Health.

With live births (LB), hospitals were the main source of information (51.5%), followed by registry offices (42.2%). A total of 3.3% of LBs were found among records of social government programs but were not found in registry offices (Table 1).

In Table 2, we present the estimated parameters of the log-log models with the live birth, total death, and infant death correction factors as the response variables. For the live birth correction factor, a significant association (p < 0.0001) was found between the correction factor calculated among sampled municipalities and the indicator of SINASC completeness (logarithm of ***R***_***LB***_). For the total death correction factor, the results of the log-log model indicate a high and significant correlation (p < 0.0001) between the estimated correction factors in the proactive search and the indicator of SIM completeness (logarithm of ***R***). In relation to infant deaths**,** besides the positive association with the total death correction factor, we found an additional significant effect of the variable ***R***_***IM***_, that is, as the IMR moves away from the median among municipalities with adequate information, the correction factor increases. The multiple correlation coefficient was 0.777 (p < 0.0001).

**Table 2:** Log-log models fitted to live births, total and infant death correction factors*, Brazilian regions, 2008

**Model**	**Coefficients**			
	**B**	**Std. error**	**t**	**Sig.**
Response variable: live birth correction factor*				
(Constant)	0.059	0.007	8.327	0.000
Logarithm of ***R***_***LB***_	−0.473	0.038	−12.352	0.000
Multiple Correlation Coefficient (R): 0.739 (<0.0001).				
Response variable: total death correction factor*				
(Constant)	0.026	0.006	4.123	0.000
Logarithm of ***R***	−0.955	0.018	−52.133	0.000
Multiple correlation Coefficient (R): 0.977 (<0.0001).				
Response variable: infant death correction factor*				
(Constant)	0.036	0.022	1.609	0.111
Logarithm of the total death correction factor	1.058	0.089	11.948	0.000
Logarithm of ***R***_***IM***_	−0.076	0.035	−2.157	0.033
Multiple correlation coefficient (R): 0.777 (<0.0001).				

*Obtained in the sampled municipalities of the proactive search.

Table 3 shows death and LB correction factors by level of reporting completeness. As expected, the death underreporting correction factor increases as the age-standardized mortality rate decreases. Infant death correction factors are always higher than those estimated for the total number of deaths. On the other hand, the LB correction factors are much lower than those estimated for deaths, even among municipalities with inadequate LB information.

**Table 3:** Municipal deaths and live birth correction factors by level of reporting, Brazil, 2008

Category of ASMR* (per 1,000 inhabitants)	**Death correction factor**			
	**Infants**	**Total**	**R**_**LB **_category**	**LB correction factors**
<2	7.17	3.40	<0.5	2.30
≥2 to <3	3.17	2.18	≥0.5 to <0.6	1.74
≥3 to <4	2.11	1.51	≥0.6 to <0.7	1.40
≥4 to <5	1.52	1.20	≥0.7 to <0.8	1.19
≥5 to ≤5.5	1.24	1.06	≥0.8 to <0.9	1.08
≥5.5	1.10	1.03	≥0.9	1.05

*ASMR: age-standardized mortality rate using the Brazilian population, 2008, as the standard.

**R_**LB**_: ratio between reported and estimated live births**.**

After the correction factors were applied to all municipalities for the period 2000–2010, infant mortality rates were estimated according to the age-standardized mortality rate category and for Brazil (Table 4). In the period 2000–2010, the IMR declined from 26.1 to 16.0 deaths per 1,000 LB, with an annual reduction rate of 4.7%. However, for the municipalities with less reliable information, the IMR was greater than 40 deaths per 1,000 LB between 2000 and 2009, with a reduction rate of only 1.7% per year from 2000 to 2010.

**Table 4:** Infant mortality rate (IMR), municipality percentage, and resident population percentage by ASMR* level, Brazil, 2000-2010

**ASMR category (per 1,000 inhabitants)**	**2000**	**2001**	**2002**	**2003**	**2004**	**2005**	**2006**	**2007**	**2008**	**2009**	**2010**
IMR											
<3	48.1	48.3	45.9	48.3	47.5	45.3	45.8	43.8	42.6	44.5	38.6
≥3 and <5.5	28.9	28.0	25.9	25.8	24.0	22.9	22.0	20.8	20.1	19.4	18.8
≥5.5	22.1	21.1	20.5	19.5	19.0	17.9	17.1	16.4	15.8	15.1	14.6
Total	26.1	24.9	23.4	22.5	21.5	20.4	19.6	18.6	17.7	16.8	16.0
% municipalities											
<3	13.4	11.0	7.8	6.0	4.9	4.6	4.6	4.3	3.3	2.7	2.3
≥3 and <5.5	57.0	58.9	59.8	58.7	57.9	55.5	55.8	52.5	55.4	51.8	49.2
≥5.5	29.6	30.1	32.4	35.4	37.3	39.9	39.6	43.2	41.2	45.5	48.4
% population											
<3	5.5	4.2	3.0	2.4	2.0	1.9	1.8	1.7	1.3	1.1	0.8
≥3 and <5.5	32.0	32.5	34.0	31.9	34.5	34.9	36.0	34.7	32.1	28.6	25.9
≥5.5	62.5	63.2	63.0	65.7	63.5	63.2	62.2	63.6	66.6	70.3	73.2

*ASMR: age-standardized mortality rate using the Brazilian population as the standard in each year of the period 2000–2010.

With regard to the reporting of vital information, the results shown in Table 4 also demonstrate the progress achieved. Among all Brazilian municipalities, 13.4% had poor mortality data (ASMR < 3 per 1,000 inhabitants) in 2000. This percentage decreased to 2.3% in 2010. Additionally, municipalities with a good level of mortality information (ASMR ≥5.5 per 1,000 inhabitants) comprised 62.5% of the Brazilian population at the beginning of the period, which improved to 73.2% at the end of the decade. It is worth noting that in 2010, only 0.8% of the Brazilian population lived in municipalities with very unreliable death information.

The results shown in Table 5 indicate significant increases in the completeness of death and live birth information in the North and Northeast regions, which have been historically characterized by underreporting of vital data. In the North, the completeness of LB and death reporting reached 90% and 85%, and in the Northeast, 93% and 89%, respectively. The advances in the regions with the greatest reporting problems are reflected nationally: in Brazil, the completeness of LB and death information increased by approximately 3.5% per year. Nevertheless, underreporting of infant deaths remained above 20% in the North and Northeast regions in 2010.

**Table 5:** Completeness of vital information reporting and IMR estimates (reported and corrected data), Brazilian regions, 2000-2010

**Region**	**Completeness (%)**	**Completeness (%) of vital information reporting**										
		**2000**	**2001**	**2002**	**2003**	**2004**	**2005**	**2006**	**2007**	**2008**	**2009**	**2010**
North	Infant Deaths	60.2	61.0	64.5	64.4	66.4	66.6	65.5	67.3	71.1	71.8	74.1
	Total Deaths	75.3	76.6	79.0	79.0	80.4	80.4	80.3	81.7	83.4	84.4	85.4
	LB	81.3	83.7	86.4	88.3	89.6	90.2	91.0	91.5	90.7	90.5	90.5
Northeast	Infant Deaths	60.8	65.3	69.4	71.1	71.6	72.3	72.2	72.9	74.8	76.6	78.1
	Total Deaths	79.6	82.0	83.9	84.9	85.1	85.4	85.5	86.1	87.2	88.1	88.9
	LB	86.0	87.9	89.8	90.7	91.5	91.8	92.1	92.2	92.8	93.0	92.9
Brazil	Infant Deaths	74.1	76.1	78.5	79.4	80.1	80.6	80.3	81.1	82.7	83.8	85.0
	Total Deaths	91.0	91.7	92.5	92.9	93.1	93.2	93.2	93.5	94.0	94.3	94.4
	LB	92.5	93.4	94.3	94.9	95.4	95.5	95.6	95.7	95.8	96.0	96.0
**Region**	**IMR estimates (per 1,000 LB)**	**2000**	**2001**	**2002**	**2003**	**2004**	**2005**	**2006**	**2007**	**2008**	**2009**	**2010**
North	Reported*	24.3	23.4	22.1	21.3	20.6	19.9	19.2	18.5	18.0	17.6	17.2
	Corrected**	32.8	32.1	29.7	29.3	27.8	27.1	26.8	25.3	23.1	22.3	21.0
Northeast	Reported*	25.4	24.8	23.7	22.9	21.7	20.4	19.4	18.3	17.5	16.6	16.0
	Corrected**	35.9	33.4	30.8	29.3	27.8	25.9	24.8	23.2	21.8	20.3	19.1
Brazil	Reported*	20.8	20.2	19.4	18.7	17.9	17.1	16.4	15.7	15.2	14.6	14.2
	Corrected**	26.1	24.9	23.4	22.5	21.5	20.4	19.6	18.6	17.7	16.8	16.0

*reported number of infant deaths x 1,000/reported number of live births.

**corrected number of infant deaths x 1,000/corrected number of live births.

After correcting the vital statistics, there is a pronounced reduction in infant mortality in all regions of the country in the period 2000–2010 (Table 5). The highest rate of decrease occurred in the Northeast region (5.9% per year), followed by the North region (4.2), the Southeast (4.0%), the South (3.9%), and Center-West (3.2%). Comparing IMR trends using informed and corrected vital data shows that the slopes in the North and Northeast regions are accentuated as a result of improvements in completeness of death information (Figure 1).

**Figure 1:**
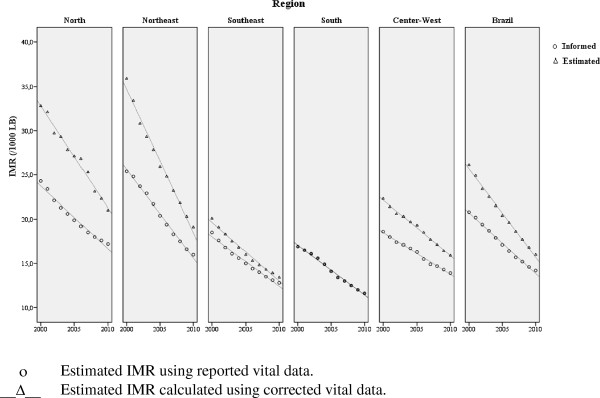
Infant Mortality Rate (IMR) estimates using reported and corrected data, Brazilian regions, 2000-2010.

## Discussion

The growing interest in measuring infant mortality, not only as a health indicator but also as a marker of human development, has encouraged the use of demographic methods for its estimation. For countries that lack vital information reporting systems or have insufficient information to estimate mortality indicators, methods have increasingly adopted estimation procedures based on complete birth histories collected from women of reproductive age in household surveys [19-21].

However, the quality of estimates based on household surveys strongly depends on the sample size, the design of the study, and the way the survey is carried out. Due to these limitations, the indirect estimates cannot be provided in small population areas, making it difficult to assess the performance of health programs focused on maternal and child health at the local level [22]. In this context, interest in continuous reporting systems has been renewed [23,24].

In Brazil, the proactive search for deaths and LBs has been adopted as an approach to detect vital events not reported to the MoH [10]. A study conducted during the early 2000s revealed a high number of infant death omissions in poor areas of the North and Northeast regions [17]. In Mexico, the active search approach was used to find births and deaths in a sample of municipalities with a low human development index in 2007–2008. The study showed a significant underreporting of deaths of children less than 5 years of age, and evidenced that more than 60% of live births did not have birth certificate [25].

The results of the present study emphasize the potential of active search procedures for identifying deaths and LBs not reported to the MoH. The high percentage of vital events found in official sources, such as hospitals and registry offices, shows problems with information systems implementation. On the other hand, the considerable participation of cemeteries (official and unofficial) and funeral houses in the detection of underreported deaths highlights the importance of searching alternative sources.

But the major contribution of this study was the estimation of live birth and death underreporting in all Brazilian municipalities, states, and regions. Because we used a probabilistic sample of municipalities stratified by the adequacy of vital information, it was possible to estimate correction factors according to municipal levels of death and LB reporting. This approach allowed vital information to be corrected by state and macro-geographical region in the period 2000–2010, by applying correction factors according to the level of reporting completeness.

Despite the great advantages of the process of active search in correcting information and detecting problems at the local level, the limitations of this method should also be pointed out. The search for vital events depends on several factors, such as the extent of the area and the number of health facilities, registries, and other sources of information to be covered. In the case of large cities and capitals, the material to be investigated is labor-intensive, but loss of information does not greatly affect the results. However, when it comes to small population municipalities, research is easier, but the loss of an event can significantly affect the estimates. To minimize these problems, the correction factors found in the sampled municipalities were not used individually to correct the information, but only to support statistical modeling.

Regarding IMR trends in the period 2000–2010, after correcting the vital statistics, a continuing and significantly decreasing trend was found, in contrast to the indirect estimates based on the National Household Sample Survey, which indicated a stabilization trend after 2005. However, the updated IMR indirect estimate based on the 2010 Demographic Census [26] is very close to the 2010 IMR estimate depicted here.

As to the progress in vital information reporting in Brazil, the growing proportion of the population that lives in municipalities with a satisfactory level of mortality information is one of the most noteworthy findings. Significant increases in the completeness of vital information were observed primarily in the North and Northeast regions, as has been previously noted by other authors [27].

Due to the increase in mortality reporting over the decade, the comparison of the infant mortality rate calculated with corrected and informed vital information showed changes in the pattern in the North and Northeast regions, with more pronounced decreasing trends evident after correcting the data. Given that the fourth Millennium Development Goal is to reduce the childhood mortality rate by two-thirds between 1990 and 2015, changes in the trends of indicators triggered by the estimation methods are particularly important because they can result in different conclusions in terms of both progress and achievement of the goal [28].

The estimated infant mortality rates in 2000–2010 indicate a continuous reduction, with an annual decreasing rate of 4.7% in Brazil, higher than the rate of 4.4% required for achieving the Millennium Development Goal of a two-thirds reduction in 25 years [29]. The Northeast region has the highest rate of reduction, 5.9% per year between 2000 and 2010, followed by the North region (4.2%), narrowing the regional inequalities that have been observed for several decades. The largest reduction in infant mortality in the Brazilian regions with lower levels of socioeconomic development undoubtedly reflects the benefits related to the expansion of primary health care. This expansion has resulted in increased access to basic health care services for these populations, which is important to the health of children and women before, during, and after pregnancy [30-32].

Nevertheless, a persistent challenge is the inequality of information on vital events, which compromises the assessment of factors that affect infant mortality in deprived areas [33-35]. It is worth noting that the municipalities with unreliable death information show the highest infant mortality and the lowest decreasing rate in the period 2000–2010.

## Conclusion

In Brazil, we concluded that it is necessary to assess the context in which data are produced, the municipality. The proactive search made it possible to identify problems in the implementation of vital information systems at the municipality level, to propose interventions to reduce local irregularities, and to develop a method to correct vital statistics in the 2000s. The investigation of death conditions among infants and the routine use of active search procedures by local health services, as recommended by the current health surveillance policies, should be implemented in problematic municipalities [12,36] to continue the process of reducing infant mortality in Brazil and to support pragmatic and viable alternatives in different social contexts.

## Competing interests

The authors declare that they have no competing interest.

## Authors’ contributions

CLS designed the study, participated in data analysis, and drafted the paper. PGF participated in the study design and coordinated research in the Northeast Region. PRBSJ coordinated research in the North Region and contributed to data analysis and result interpretation. WSA participated in data analysis. OLMN participated in study design, discussion of results, and writing text. All authors read and approved the final manuscript.
